# Psychometric properties of the workplace psychologically violent behaviors-WPVB instrument. Translation and validation in Greek Health Professionals

**DOI:** 10.3934/publichealth.2019.1.79

**Published:** 2019-02-27

**Authors:** Aristotelis Koinis, Emmanouil Velonakis, Chara Tzavara, Foteini Tzavella, Styliani Tziaferi

**Affiliations:** 1 University of Peloponnese, Department of Nursing, Laboratory of Integrated Health Care, Sparta, Greece; 2 National and Kapodistrian University of Athens, Faculty of Health Sciences, Athens, Greece; 3 Medical School of Athens

**Keywords:** health professionals, instrument, mobbing, nurses, psychometrics

## Abstract

**Background:**

Mobbing exerts severe psychological and occupational effects on the victim. This study aims to validate the Yildirim & Yildirim's Workplace Psychologically Violent Behaviors (WPVB) instrument (2008) in the Greek language in Greece, as cultural variations may result in significantly different perceptions of mobbing.

**Methodology:**

A translation process of the WPVB questionnaire scale was followed from the English to the Greek version and a review by a team of experts for its content validity took place, as well. Principal component analysis took place and the Cronbach's index was 0.95. The cross sectional, quantitative study was performed in 1536 health professionals (HPs), working in 11 public hospitals for at least one year with response rate of 76.8%.

**Results:**

Factor analysis revealed two factors, and 31-item construct, compared to the four factors and the 33-item construct of the original version of the tool. All items were found to have a statistically significant correlation (*p* < 0.001). Median score was 0.48. Whereas 25% of answers score was above 1.00, thus suggesting significant mobbing in around 25% of HPs. Association of WPVBs subscales with sex and occupation are had lower values in women as compared to men. Lower scores on “Attack on personality” and “Total mobbing” score were recorded in nurses as compared to doctors. Doctors had lower scores on “Individual's isolation from work” as compared to administrative personnel, while had greater scores on “Individual's isolation from work” as compared to technicians. Nurses had significantly lower scores on “Attack on professional status”, “Individual's isolation from work”, “Direct attack” and “Total mobbing” score as compared to administrative personnel.

**Conclusions:**

The study highlights that the phenomenon of mobbing exists in Greek HPs regardless of age, gender, level of study and negatively affects their lives. Focusing on improving this area, is expected to promote occupational health and safety of these workers.

## 1. Introduction

The phenomenon of mobbing involves employees “ganging up” on a target employee and subjecting him or her to psychological harassment [1]. This “mobbing” behavior exerts severe psychological and occupational effects on the victim. In Greece, it seems that the phenomenon of moral harassment exists in all work environments including the health sector [2]. A study on the effect of mobbing on the professional life of nurses in seven Greek hospitals showed that nurses, men, and women (71%), had been victims of moral harassment during the past year and had psychosomatic symptoms (anxiety 54.3%, headaches 52%, denial of work 28%, depression 16.3% etc), [3]. Also, 22.8% of the respondents stated that they had been harassed at some time by a colleague during working in the hospital. Of the victims of moral harassment, only 38.2% appealed for help. The majority (80%) said that their personal life [3] had been affected. In a study by Yildirim and Yildirim [4], on the moral harassment of nurses in public and private healthcare facilities by their heads and colleagues that lead to mental and physical health effects, it was found that the vast majority (85.6%) were victims of moral harassment over the last 12 months. A research in Cyprus among the Health-care professionals for the prevalence and forms of workplace bullying has shown that 135 employees (45.6%) were exposed to at least one bullying behavior at work within the previous 12 months, whereas 9.9% were exposed to at least one bullying behavior at least once weekly within the previous 12 months [5]. In another similar study by Sahin et al. [6], registered doctors of Turkish origin who worked in private hospitals and as auxiliary staff at university institutions reported that they were more exposed to moral harassment behaviors (87.7%) than permanent medical staff. A study in Taiwan by Pai and Lee [7] in nurses in public hospitals investigating the risk factors and the consequences of physical and psychological violence for their mental health showed that of 521 participants, 102 (19.6%) reported that they had been subjected to physical violence, 268 (51.4%) had suffered verbal abuse, 155 (29.8%) had experienced/threats and 67 (12.9%) had sexual harassment. A recent study by Erdogan & Yildirim [8] for the exposure of healthcare professionals' to mobbing behaviors reported that the rate of exposure to one of the sub-dimensions of mobbing scale at least once in the last year was 66.4% for isolation, 71.8% for attack on professional status, 78.1% for attack on personality, and 28.4% for direct negative behaviors. Females as compared with males and participants with low income as compared to high income were more exposed to mobbing. Postgraduate participants less commonly suffered from mobbing. The nurses as compared with doctors were more exposed to mobbing and the individuals with an occupational experience of >10 years were more exposed to mobbing.

In a recent study in Turkey about mobbing behaviors encountered by nurses and their effects on nurses, showed that among the nurses participating in this study, 62.2% stated that they encountered mobbing behaviors in the workplace once or for more times over the last twelve months. The most frequent mobbing behaviors experienced by the nurses were in the form of “Attack on Professional Status” (67.7%). [9]

Although there is a small number of studies in Greece-[10]–[14] for the impact of mobbing on healthcare providers, according to studies from abroad, it is imperative to validate the specific tool to measure psychological violence and behaviors that will help to investigate these effects. According to the effects of mobbing behaviors on healthcare providers, recent studies have shown that they cause many mental and physical health problems and have a negative impact on their quality of life. In the study by Durmus et al. [9], mobbing behaviors in the workplace often affected the nurses psychologically.

In a study conducted with nurses in Turkey, Yildirim [15] found that reactions to mobbing behaviors tended to be physical disorders such as fatigue and stress, followed by over-eating or poor appetite and headaches. Silva Joao and Saldanha Portelada (2016) reported similar results, too. In their study, the nurses who were exposed to mobbing frequently experienced anxiety, insomnia, restlessness, failure, distrust feelings and impaired concentration. Therefore, it is consistently difficult for an unhappy, sleepless, anxious and distracted member of the nursing profession with low self-esteem and profoundly disturbed wellbeing state to carry out the nursing process successfully and to provide quality services [16],[17]. When the effect of mobbing on the performance of the nurses was evaluated, the nurses marked statements such as decreased commitment to work, lack of concentration and making more mistakes. Thus, the diminished work efficiency or motivation of a nurse who is suffering from mobbing directly leads to poor quality of care for patients/healthy individuals. A decrease in patient care quality may cause many risk factors such as prolongation of hospital stay by adversely affecting patients' health [16],[17]. A 117 descriptive study in Turkey by Ekici and Beder [18] assessed the prevalence of intimidation in the workplace of 201 doctors and 309 nurses working at a university hospital in Turkey, as well as the effects of the phenomenon. The study showed that psychological violence in the workplace has a significant effect on the development of the depressive disorder of both doctors (27%) and nurses (33%). The study of Malinauskiene and Einarsen [19] investigates intimidation in the workplace and the symptoms of post-traumatic anxiety disorder in family doctors in Lithuania. The prevalence of post-traumatic symptoms was 15.8%, quite high for general practitioners. A study in Iran by Najafi and his colleagues [20] investigated the perceptions and experiences of Iranian nurses about past experiences of violence against them, as well as the implications of workplace violence by patient relatives, colleagues and their directors/supervisors. In addition to the negative impact on the personal and family lives of the nurses identified, workplace violence can lead to lower quality of care for patients and a negative attitude what the nursing profession [20]. A very recent qualitative study by Hassanjhani et al. [21] took place in Iran on the consequences of mobbing in the lives of 16 nurses working in the emergency departments of five hospitals in Azerbaijan. It has been found that the practice of violence and harassment in the workplace can lead to consequences that negatively affect their quality of life in general. In particular, it can lead to psychological and psychiatric disorders, organic diseases, questioning their professional integrity, and social stigma and withdrawal.

People who suffer from ethical and psychological violence/harassment at work have increased cooperative difficulties, have reduced stress resistance, and feel physical discomfort [22].

The serious consequences of the mobbing at the workplace are not limited to the victim itself but extend to the body/service where such phenomena occur. The organizational and team productivity and efficiency of public services are undermined [23]. Negative effects for the HPs have significant impact both in their professional and personal lives, contributing to a negative climate in the organization as whole. Mobbing can be responsible for a wide variety of psychological and social problems, which can include absence from the workplace, lower productivity at work, burnout, stress, anxiety and even depression [24]. Also, the critical review of Fountouki et al. [25] about the effect of anxiety in nursing staff has shown that nurses when they are in situations under heavy stress that has a negative impact on their professional lives, such as insufficient work recourses, dissatisfaction with psychosocial work environment, poor communication with superiors, lowering levels of education achieved and pay, split-shifts and prolonged night shifts, high demanding tasks, verbal abuse, mobbing and antagonistic attitudes in work place and poor organization at work [25].

On the other hand, a well-developed reliable taxonomy of workplace bullying has not yet been established and workplace bullying has been categorized often with little scientific evidence. Nevertheless, the work of Rodriguez-Carballeira et al. [26] is a considerable step towards this direction, placing emphasis, on emotional and physical isolation, which is considered as a separate category. The development of a reliable and widely accepted taxonomy could play a critical role in instrument development or refinement and allow the reliable measurement and description of this phenomenon.

Although mobbing is frequent, one only validated instrument has been used to measure it in Greece. Besides The Negative Acts Questionnaire (NAQ-22), which is measuring perceived exposure to workplace bullying in teachers, and the Greek version of Leymann Inventory of Psychological Terror (LIPT) instrument, which was validated by Zachariadou et al. [5], no other instrument exists in our country for the measurement of psychological violence that health professionals are exposed to in the workplace. The instrument proposed by Yildirim and Yildirim [4] comprises a wide range of mobbing behaviors, it is designed especially for nurses and has been validated in the Turkish nurse population. The purpose of the present study was to validate this instrument in Greek language in Greece, as cultural variations may result in significant different perceptions of mobbing.

### 1.1. The importance of the study

“Mobbing” or “harassment syndrome” is a reality for most workers. It is observed—if not all in most professions—and in most workplaces, and thus in that of Hospitals, which in any case on its own, is entrusted owing to its special character. Mobbing does not seem to have been studied in depth and wide enough to cover all occupations in all.

The phenomenon has been alarming in our country, even though extensive and in-depth investigations have not been carried out as opposed to foreign countries. The first scientifically substantiated studies [27]–[31] show that this phenomenon is detrimental to the physical and mental health of workers and, by extension, to their families and is one of the most important causes of abandonment [27]–[31]. Given that:

- “the actual dimension of mobbing is not yet widely known,
- it has a significant impact on the mental and physical health of the employees and also affects their family, professional and social life,
- it is an interesting scientific subject, which needs more study and exploration and finally it has not been studied enough in our country's healthcare professionals and we believe it is necessary to carry out research to study the moral harassment of HP's and how it affects their quality of life”[32]–[34].

The present study through the questionnaire accurately captures the magnitude of the problem in the working sector of health services in Greece, behaviors of moral harassment, forms of harassment as well as investigate who these behaviors come from, at this workplace. Finally, it helps to tackle the phenomenon through performing appropriate intervention tools and procedures (information and prevention programs) by both mental health professionals and decision-makers in the field of inter-disciplinary teamwork of occupational health and safety services in Greece.

## 2. Materials and methods

The study's convenience sample consisted of 1536 HPs, working in 11 public hospitals for at least one year, in two health prefectures in Greece. The WPVB instrument used in this study, was initially comprised of 33 items and according to its inventors, shows a four-factor construct. A) Individual's isolation from work, B) Attack on professional status, C) Attack on personality, D) Direct attack. Within the Greek version of this instrument, 11 items are related to “individual's isolation from work”, 9 items related to “attack on professional status”, 9 items related to “attack on personality” and 4 items related to “direct attack”, other than “direct attack”, were shown to have quite high levels of internal consistency. The Greek version of WPVB questionnaire has been proven reliable and valid, with a Cronbach's a found to be 0.93.

HPs were asked to mark the frequency within the last 12 months that they had faced items on the given list they were given of workplace antagonistic and unethical behaviors that have had a negative effect on their work performance and to mark also, by whom every behavior they had been exposed to had come from (supervisor, coworker, subordinate or other). A six-point Likert type scale was used for the determination of frequency of mobbing behaviors as follows: 0 = I have never faced, 1 = I have faced once, 2 = I face this sometimes, 3 = I have faced several times, 4 = I frequently face this, 5 = I constantly face this. Individuals who receive a score from the scale divided by the number of items finally included in the instrument (total score/31), that is one or greater, can be said to have faced intentional workplace mobbing behaviors. Questions no.13 and 33, (B1 and D1 in the original version respectively) were excluded from the analysis since they were answered by four individuals only.

### 2.1. Statistical analysis

Continuous variables are presented with mean and standard deviation (SD) or with median and inter-quartile range (IQR). Qualitative variables are presented with absolute and relative frequencies. In order to evaluate the construct validity of the questionnaire, a confirmatory factor analysis (CFA) with maximum likelihood procedure was performed. The variance of the latent constructs was fixed at one during parameter estimation. The fit of the CFA model was assessed using the chi-square (χ^2^), the comparative fit index (CFI), the goodness of fit index (GFI) and the root mean square error of approximation (RMSEA) [35]. For the CFI and GFI indices, values close to or greater than 0.95 are taken to reflect a good fit to the data [36]. RMSEA values of less than 0.05 indicate a good fit and values as high as 0.08 indicate a reasonable fit [36]. Also, a non-significant chi-square statistic indicates a good fit, but chi-square is usually sensitive to sample sizes and usually significant for large sample sizes [35]. The internal consistency of the questionnaire was analyzed with Cronbach's α. Reliability equal to or greater than 0.70 was considered acceptable. Spearman correlations coefficients were used to explore the association among the WPVB subscales and the association of WHO-Bref, GHQ-28 and Social Support dimensions with WPVB subscales in terms of convergent validity. The correlation coefficient between 0.1 and 0.3 was considered low, between 0.31 and 0.5 moderate and over 0.5 was considered high. In terms of discriminative validity, the WPVBs subscales were compared according to sex using Mann-Whitney tests, while Kruskal-Wallis tests were used for comparisons according to the occupation. Bonferroni correction was used in case of multiple comparisons for occupation. P values reported are two-tailed. Statistical significant level was set at 0.05 and analysis was conducted using SPSS 22.0 and AMOS (SPSS, Chicago, IL, USA) Statistical Software.

## 3. Results

Participants were 528 men and 1008 women (N = 1536) with a mean age of 39.2 years (SD = 10.3 years). Sample characteristics are presented in Table 1. 47.3% of the participants were married and 56.5% had children. 23.2% were doctors, 48.4% were nurses, 16.4% were administrative personnel, 3.7% technicians and 8.3% other health professionals, 23.7% of the sample reported as having a health problem.

Descriptive statistics for the WPVB items are shown in Table 2. Items 1, 2 and 11 had a median value equal to 1.

A CFA was conducted to estimate if the model fitted the data well. The CFA indicated an adequate fit of the four-factor model (RMSEA = 0.078, CFI = 0.961 and GFI = 0.932). None of the item cross-loadings exceeded the item loadings on the intended latent construct. The chi-square test of the model was significant as expected (*p* < 0.05).

Corrected item-total correlations and Cronbach's and if an item was deleted per factor are presented in Table 3. All corrected item-total correlations were high and internal consistency reliability was accepted with Cronbach's alpha equal to 0.90 for Attack on personality, 0.92 for Attack on professional status, 0.93 for Individual's isolation from work and 0.78 for Direct attack. Cronbach's alpha for all questionnaire was equal to 0.87.

The inter-correlations of the WPVB subscales are shown in Table 4. All subscales were significantly and positively correlated with each other and the correlations were high.

Correlations of WPVBs subscales with WHO-Bref subscales were all negative and significant (Table 5). Also, WPVBs subscales were significantly and positively correlated with all GHQ-28 dimensions. Furthermore, all WPVBs dimensions were significantly and negatively correlated with all Social Support dimensions.

Association of WPVBs subscales with sex and occupation are presented in Table 6. All subscales had lower values in women as compared to men.

Lower scores on “Attack on personality” and “Total mobbing” score were recorded in nurses as compared to doctors. Doctors had lower scores on “Individual's isolation from work” as compared to administrative personnel, while had greater scores on “Individual's isolation from work” as compared to technicians. Nurses had significantly lower scores on “Attack on professional status”, “Individual's isolation from work”, “Direct attack” and “Total mobbing” score as compared to administrative personnel. Technicians had lower score on “Individual's isolation from work” as compared to nurses, while administrative personnel had greater scores on “Attack on professional status”, “Individual's isolation from work” and “Total mobbing” score as compared to technicians. Also, technicians had lower scores on “Attack on professional status” and “Individual's isolation from work”, as compared to others.

**Table 1.** Sample characteristics.

	N (%)	Mean (SD)
Sex		
Men	528 (34.4)	
Women	1008 (65.6)	
Age, mean (SD)		39.2 (10.3)
Educational Status		
At most High school/College	341 (22.3)	
Technical university	536 (35.1)	
University	316 (20.7)	
MSc/PhD	336 (22.0)	
Marrital Status		
Married	725 (47.3)	
Having children	851 (56.5)	
Living		
* Alone*	339 (23.0)	
* With others*	1137 (77.0)	
Occupation		
Doctors	348 (23.2)	
Nurses	726 (48.4)	
In administration	246 (16.4)	
Technicians	56 (3.7)	
Other	124 (8.3)	
Working		
No	76 (5.0)	
Yes	1446 (95.0)	
Residence		
Athens	786 (51.2)	
Out of Athens	750 (48.8)	
Health status		
Very bad	53 (3.5)	
Bad	71 (4.6)	
Neither bad nor good	366 (23.8)	
Good	697 (45.4)	
Very good	349 (22.7)	
Having health problem	364 (23.7)	

**Table 2.** Descriptive statistics for the WPVB items.

	Mean (SD)	Median (IQR)
item 1	1.51 (1.43)	1 (0–3)
item 2	1.17 (1.35)	1 (0–2)
item 3	1.17 (1.40)	0 (0–2)
item 4	0.81 (1.38)	0 (0–1)
item 5	0.83 (1.33)	0 (0–2)
item 6	0.45 (1.04)	0 (0–0)
item 7	1.22 (1.53)	0 (0–2)
item 8	1.01 (1.27)	0 (0–2)
item 9	1.15 (1.38)	0 (0–2)
item 10	0.54 (1.02)	0 (0–1)
item 11	1.09 (1.24)	1 (0–2)
item 12	0.67 (1.08)	0 (0–1)
item 13	0.57 (0.99)	0 (0–1)
item 14	0.59 (1.03)	0 (0–1)
item 15	0.29 (0.78)	0 (0–0)
item 16	1.23 (1.45)	0 (0–2)
item 17	1.04 (1.46)	0 (0–2)
item 18	0.84 (1.20)	0 (0–2)
item 19	0.59 (1.08)	0 (0–1)
item 20	0.36 (0.97)	0 (0–0)
item 21	0.51 (1.07)	0 (0–0)
item 22	0.68 (1.29)	0 (0–1)
item 23	0.78 (1.29)	0 (0–2)
item 24	0.43 (1.01)	0 (0–0)
item 25	0.88 (1.24)	0 (0–2)
item 26	0.47 (1.09)	0 (0–0)
item 27	0.28 (0.83)	0 (0–0)
item 28	0.38 (0.93)	0 (0–0)
item 29	0.59 (1.13)	0 (0–1)
item 30	0.39 (0.99)	0 (0–0)
item 31	0.69 (1.22)	0 (0–1)
item 32	0.42 (0.95)	0 (0–0)
item 33	0.25 (0.73)	0 (0–0)

**Table 3.** Corrected Item-Total Correlations, internal consistency reliability and descriptive statistics of the WPVB factors.

	Corrected Item-Total Correlation	Cronbach's Alpha if Item Deleted	Cronbach's a	Mean (SD)	Median (IQR)
*Attack on personality*					
item 1	0.69	0.89	0.90	8.78 (8.79)	6 (0–14)
item 2	0.68	0.89			
item 3	0.76	0.88			
item 4	0.67	0.89			
item 6	0.52	0.90			
item 7	0.73	0.89			
item 8	0.79	0.88			
item 9	0.74	0.89			
item 15	0.49	0.90			
*Attack on professional status*					
item 5	0.71	0.91	0.92	6.61 (8.16)	4 (0–10)
item 10	0.76	0.91			
item 11	0.76	0.91			
item 12	0.76	0.91			
item 13	0.78	0.91			
item 14	0.79	0.91			
item 16	0.67	0.92			
item 21	0.75	0.91			
item 29	0.59	0.92			
*Individual's isolation from work*					
item 17	0.66	0.93	0.93	7.16 (9.94)	3 (0–10)
item 18	0.71	0.92			
item 19	0.73	0.92			
item 20	0.53	0.93			
item 22	0.77	0.92			
item 23	0.76	0.92			
item 24	0.68	0.93			
item 25	0.77	0.92			
item 26	0.76	0.92			
item 30	0.75	0.92			
item 31	0.77	0.92			
*Direct attack*					
item 27	0.58	0.73	0.78	1.33 (2.69)	0 (0–2)
item 28	0.67	0.69			
item 32	0.64	0.70			
item 33	0.47	0.78			

**Table 4.** Intercorrelations among WPVBs subscales.

	Attack on personality	Attack on professional status	Individual's isolation from work	Direct attack	Total mobbing score
Attack on personality	1.00				
Attack on professional status	0.79	1.00			
Individual's isolation from work	0.74	0.82	1.00		
Direct attack	0.64	0.63	0.71	1.00	
Total mobbing score	0.92	0.92	0.91	0.72	1.00

Note: all correlation coefficients were significant (*p* < 0.001).

**Table 5.** Correlation of WPVBs subscales with WHO-Bref, GHQ-28 and Social Support subscales.

	Attack on personality	Attack on professional status	Individual's isolation from work	Direct attack	Total mobbing score
*WHO-Bref*					
Total score	−0.14***	−0.08**	−0.12***	−0.17***	−0.11***
Physical health	−0.20***	−0.13***	−0.13***	−0.17***	−0.15***
Psychological health	−0.17***	−0.14***	−0.14***	−0.14***	−0.15***
Social relationships	−0.19***	−0.13***	−0.15***	−0.17***	−0.16***
Enviroment	−0.19***	−0.11***	−0.12***	−0.16***	−0.13***
*GHQ-28*					
Somatic symptoms	0.14***	0.16***	0.15***	0.17***	0.15***
Anxiety/insomnia	0.17***	0.19***	0.19***	0.19***	0.19***
Social dysfunction	0.08**	0.13***	0.08**	0.13***	0.10***
Severe depression	0.06*	0.15***	0.12***	0.13***	0.10***
Total score	0.14***	0.20***	0.17***	0.20***	0.18***
*Social Support*					
Total support	−0.18***	−0.14***	−0.18***	−0.20***	−0.17***
Support from family	−0.19***	−0.13***	−0.17***	−0.18***	−0.17***
Support from friends	−0.16***	−0.14***	−0.16***	−0.17***	−0.15***
Support from significant other	−0.18***	−0.13***	−0.16***	−0.19***	−0.17***

Note: *p < 0.05; **p < 0.01; ***p < 0.001

**Table 6.** Association of WPVBs subscales with sex and occupation.

	Attack on personality	Attack on professional status	Individual's isolation from work				Direct attack		Total mobbing score	
	
	Mean (SD)	Median (IQR)	Mean (SD)	Median (IQR)	Mean (SD)	Median (IQR)	Mean (SD)	Median (IQR)	Mean (SD)	Median (IQR)
Sex										
Men	10.27 (9.47)	8 (1.5–18)	7.54 (8.5)	5 (0–12)	8.31 (10.81)	3 (0–13)	1.83 (3.11)	0 (0–3)	27.95 (29.82)	17 (4–47)
Women	8.13 (8.41)	6 (0–13)	6.2 (8.03)	3 (0–9)	6.66 (9.52)	2 (0–9)	1.1 (2.45)	0 (0–1)	22.1 (26.05)	15 (2–30)
P^1^	<0.001	<0.001	0.017	<0.001	<0.001					
Occupation										
Doctors^A^	9.34 (7.79)^B^	9 (3–15)	6.39 (7.17)	5 (0–10)	6.36 (7.8)^C,D^	3 (0–10)	1.32 (2.24)	0 (0–2)	23.41 (22.54)^B^	17 (6–32)
Nurses^B^	8.26 (8.84)^C^	6 (0–14)	6.23 (8.43) ^C^	3 (0–9)	6.97 (10.46) ^C,D^	2 (0–9)	1.32 (2.9) ^C^	0 (0–1)	22.78 (28.44) ^C^	14 (2–29)
Administrative^C^	9.98 (9.01)	8 (2–18)	7.76 (7.53)^D^	6 (0–12)	8.78 (9.72)^D^	5 (0–13)	1.54 (2.75)	0 (0–2)	28.07 (27.03)^D^	19 (5–44)
Technicians^D^	8.73 (10.51)	4 (0.5–12)	5.84 (10.67)^E^	1 (0–7)	6.7 (13.37)^E^	0 (0–7)	1.13 (2.85)	0 (0–0)	22.39 (36.66)	4 (2.5–27)
Other^E^	8.84 (9.86)	5.5 (0–15)	8.22 (9.22)	6 (0–13)	8.11 (10.47)	4 (0–14)	1.24 (2.53)	0 (0–1.5)	26.41 (29.03)	17 (5–41)
P^2^	0.003	<0.001	<0.001	0.049	<0.001					

Note: ^1^Mann-Whitney test; ^2^Kruskal-Wallis test; A, B, C, D, E indicates significant differences.

## 4. Discussion

According to the findings of the present study, there is no instrument in Greece for measuring health professionals' perception of workplace psychological violence inflicted on them by their managers, co-workers and/or subordinates and this is the first study conducted nationally on this subject. In addition, the Greek version WPVB Instrument's Cronbach's value was found to be high (0.93).

The NAQ-22 scale applied in the study by Karatza et al. [11] has shown that men face mobbing behavior more than women do. However, on the NAQ-22 scale concerning the distribution of roles, it is noted that women regard themselves as victims more than men do [11]. These two findings, which at first sight appear contradictory, are clarified by the difference in the responses of men and women to mobbing behavior, which is another finding of the present study. As shown in the study by Zachariadou et al. [5] women were exposed to at least one mobbing behavior more often than men within the previous 12 months. Another study by Al-Omari [37] about gender-related result showed that female nurses report 0.5-fold less physical harassment than male nurses and 1.5% more times reported having experienced verbal harassment than male nurses. A similar study in Palestine by Jaradat et al. [38] among nurses showed that male nurses reported a higher prevalence of intimidation than female nurses. Newer and older nurses reported a higher prevalence of exposure to physical and verbal aggression and intimidation. Other recent studies showed that male health professionals are experiencing mobbing behaviors to a greater extent than women [39]–[41]. According to Mantzouranis et al. [42] study of assess workplace violence in a Greek tertiary hospital, shown that verbal violence was the most common type of incident and the vast majority of employees had experienced work-related violence. Also, HPs (nurses and other health care staff), reported that feeling safer than physicians.

Two-thirds of the men and one-third of the women perceive mobbing behavior as normal. This difference of gender in terms of response to mobbing behavior also indicates the viewpoints and perceptions of individuals concerning the phenomenon. In this context, men effectively face mobbing behavior more than women do. However, women regard themselves as victims more than men do. The reason for this is that men consider such behavior to be something normal which can happen in the workplace while women find it unacceptable.

International research suggests that gender-related experiences of workplace bullying could be country-specific. Cortina et al. [43], found that American women reported more workplace bullying experiences than men did. Niedhammer et al. [44] came to the same conclusion about France, whilst Ólafsson and Jóhannsdóttir [45] found that men experienced more workplace bullying in Iceland than women did. Ortega et al. [46] found no significant differences between Danish men and women. Furthermore, Namie [47] found that the perpetrators of 62% of American men who experienced bullying were men. In another similar study by Zachariadou et al. [5] regarding the formal position of perpetrators, superiors were pointed out as mobbers by 57.5% of the overall study population. It might be assumed that professionals who have less power could be in a more vulnerable position for exposure to bullying behaviors.

Nurses in our study have experienced mobbing behaviors in a lesser degree than doctors, and mostly behaviors of attack on the personality and attack on professional status. This is in contrast to research in nurses in Turkey that showed The most frequent mobbing behaviors experienced by the nurses were in the form of “Attack on Professional Status” and this sub-scale was followed by the “Attack on Personality” [9].

Surprisingly enough, fewer than five individuals answered the items “Having physical violence used” and “Always having errors found in your work and work results”, namely the B1 & D1 items of the original instrument. Moreover, our mean values suggest considerable workplace bullying. These differences could be attributed to cultural and methodological issues. Firstly, physical violence might seem unacceptable in the nursing working environment and thus “considered out of the question”. According to Karatza et al. [11] study using NAQ-22 as a research tool, the most prevalent bullying behaviors in the nursing workplace were related to work itself (unmanageable workloads, being assigned tasks below one's level of competence) and being subjected to “anger expressed by third parties”, while “physical violence” was minimum (4.4 points within a range of 3 to 15). According to Zachariadou et al. [5] study using LIFT as a research tool, another interesting finding of this study was that 9.4% of participants reported that they were bullied by their subordinates, indicating that a managerial position does not guarantee protection from bullying. The above findings suggest that the lack of a firm leadership contributes to the manifestation of mobbing behaviors from all the levels of the hierarchy ladder of the organization. Another study by Iftikhar and Qureshi [48] in nurses reported that the form of this mobbing behavior had a negative impact on the work performance of nurses, their motivation for work, their productivity, their relationships with patients, and symptoms of depression. Zhang et al. [49] conducted a study in 28 provinces in China in 14 cities to investigate the prevalence of the phenomenon of moral harassment and the factors that affect it. 4125 questionnaires were distributed and 3004 nurses of all grades were finally involved in the survey. The results showed that 25.77% of the respondents had experienced physical violence, 63.65% verbal abuse and 2.76% sexual harassment. The study also showed that low-skilled nurses, part-time nurses, and nurses working in China's emergency and pediatric departments have low levels of tolerance in stress and are more likely to experience any form of harassment and violence. On the other hand, detection of errors might be considered as a privilege of superiors, compatible with the organizational culture in Greece, when compared to the rest of Europe some interesting findings emerge regarding the Greek values referring to working behavior. The comparisons revealed that people in Greece attribute significantly more importance to power/achievement, conformity/tradition, among other values [50]. In this working climate, it is possible that several dimensions differ from those of Yildirim and Yildirim [51]. Besides, the instrument creators in their study mentioned that their study had several limitations. Indeed, that study was only conducted with female participants in the nursing, female-dominant, profession and in the largest university hospital in Instabul, Turkey, while in our study a great range of hospitals participated [51]. This fact could also explain the considerable difference in scores between the two studies, ours being significantly higher, suggesting workplace psychological violence. Our study emphasizes the need for further validation studies in different cultural environments, in accordance with the creators of the tool, who stated that “As the tool was developed from data collected from nurses in Turkey it reflects the cultural characteristics of the society in which it was developed. For this reason, it is recommended that this instrument is tested in samples of nurses with different cultural characteristics and with different professional groups”.

The modified Greek version of the WPVB questionnaire exhibits excellent validity and reliability and emphasizes the need for developing mobbing measurement instruments culturally adjusted. In that context, special norms could be created for different populations and the phenomenon could be studied with accuracy. In our study, although the sample was large, it is not absolutely representative of the nursing population (35,420 in Greece) [52]. Some HPs due to work overload were reluctant to participate and all hospitals included are not represented proportionally in the sample. In this context, it is difficult to construct a norm for the entire nurse population. A future well-designed study, with a random and representative sample of Greek HPs, could end up with a norm for the Greek nursing population.

## 5. The implication to research and practice

The Greek version of the WPVB questionnaire is a valid and reliable instrument to measure mobbing within the Greek population. This instrument can be a valuable tool for HPs and health care providers for use in planning strategies for mobbing provision in Greece. International studies mainly in Turkey reported that this instrument helps to investigate the phenomenon of mobbing and to design appropriate hospital management strategies [4]–[8],[11]. The issue of moral harassment in Greece has not yet been thoroughly explored, but as it emerges from International Studies, it is imperative because it has a negative impact on the quality of life of health professionals and causes problems for both the worker himself and his family and work environment [10],[53]–[55]. Areas of investigation of mobbing are satisfied or dissatisfied with can be identified. This tool can be used as a basis to initiate changes in clinical practice both in a hospital and in community health care working settings.

## 6. Future research

The Greek version of the WPVB questionnaire can be used as a tool for further investigating of mobbing provided in Greece and obtaining a better understanding of the meaning of mobbing, of the dealing with the phenomenon and of the needs, and of the expectations against mobbing at health professionals. It is anticipated that the Greek version of the WPVB will contribute to further development of the research in the field of health sector service and other working fields in Greece.
